# Multi-dimensional vulnerability analysis on catastrophic health expenditure among middle-aged and older adults with chronic diseases in China

**DOI:** 10.1186/s12874-022-01630-9

**Published:** 2022-05-25

**Authors:** Wenqing Miao, Xiyu Zhang, Baoguo Shi, Wanxin Tian, Bing Wu, Yongqiang Lai, Yuze Li, Zhipeng Huang, Qi Xia, Huiqi Yang, Fan Ding, Linghan Shan, Ling Xin, Jingying Miao, Chenxi Zhang, Ye Li, Xiaodong Li, Qunhong Wu

**Affiliations:** 1 Center for Policy and Management Research, School of Health Management, Harbin Medical University, No. 157 Baojian Road, Nangang District, Harbin, 150086 Heilongjiang China; 2 Department of Economics, School of Economics, Minzu University of China, Beijing, China; 3 Department of Medicine, Jiamusi University, Jiamusi, 154007 Heilongjiang China; 4 School of Public Health and Management, Ningxia Medical University, Yinchuan, Ningxia China; 5 The First Department of General Surgery, Heilongjiang Provincial Hospital, No. 82 Zhongshan Road, Xiangfang District, Harbin, 150036 Heilongjiang China

**Keywords:** Middle-aged and older adults, Chronic diseases, Medical insurance

## Abstract

**Objective:**

Middle-aged and older adults are more likely to suffer from chronic diseases because of their particular health characteristics, which lead to a high incidence of catastrophic health expenditure (CHE). This study plans to analyse the different factors affecting CHE in middle-aged and older adults with chronic diseases, target the vulnerable characteristics, and suggest groups that medical insurance policies should pay more attention to.

**Methods:**

The data used in this study came from the 2018 China Health and Retirement Longitudinal Study (CHARLS) database. The method of calculating the CHE was adopted from the World Health Organization (WHO). The logistic regression was used to determine the family characteristics of chronic disease in middle-aged and older adults with a high probability of incurring CHE.

**Results:**

The incidence of CHE in middle-aged and older adults with chronic disease was highest in sub-poverty level families (26.20%) was lowest in wealthier level families (20.07%). Households with malignant tumours had the highest CHE incidence under any circumstances, especially if the householder had been using inpatient service in the past year. Among the comparison of CHE incidence in different types of medical insurance, the Urban and Rural Residents’ Basic Medical Insurance (URRBMI) was the highest (27.46%). The incidence of CHE was 2.73 times (95% CI 2.30–3.24) and 2.16 times (95% CI 1.81–2.57) higher among people who had used inpatient services in the past year or outpatient services in the past month than those who had not used them.

**Conclusions:**

Relatively wealthy economic conditions cannot significantly reduce the financial burden of chronic diseases in middle-aged and older adults. For this particular group with multiple vulnerabilities, such as physical and social vulnerability, the high demand and utilization of health services are the main reasons for the high incidence of CHE. After achieving the goal of lowering the threshold of universal access to health services, the medical insurance system in the next stage should focus on multiple vulnerable groups and strengthen the financial protection for middle-aged and older adults with chronic diseases, especially for patients with malignant tumours.

## Background

With advancements in civilization, the spectrum of diseases is changing. Currently, chronic diseases are the predominant cause of human illness. The World Health Organization (WHO) reported that chronic diseases kill 40 million people per year, which accounts for 70% of global deaths [1]. Moreover, WHO data revealed that seven of the ten leading causes of death in 2019 were chronic diseases, accounting for 44% of all deaths, or 80% of the top 10 [2]. Excluding Alzheimer’s or dementia, the top five chronic diseases with the highest death rates are heart disease, stroke, chronic lung disease, malignant tumours, and diabetes. However, long courses and high costs of treatment for chronic diseases place a heavy economic burden on middle-aged and older adults [3]. Simultaneously, the proportion of middle-aged and older adults in the total population is increasing. According to data from the World Population Prospects 2019, the United Nations predicts that by 2020, the global population aged 45 years and above will reach 2.362 billion, accounting for 30.31% of the total population [4].

Moreover, in recent years, chronic diseases have become a significant public health problem impacting China’s economic and social development. According to survey data from 2015, the number of deaths and the disease burden caused by chronic diseases accounted for 86.6% and nearly 70% of China’s total deaths and total disease burden, respectively [5]. Moreover, the WHO reported in 2015 that the direct medical costs of chronic diseases in China exceeded $500 billion. If no effective measures are taken to curb this, then rapid ageing may increase the economic burden of chronic diseases by 40% by 2030 [1]. Furthermore, the burden of chronic diseases is not only heavy for countries, but also for individuals. For example, Li Liu et al. showed that the average hospitalization cost for diabetic patients was more than twice that of non-diabetic patients [6].

China’s medical insurance system is an important measure to reduce the economic burden of chronic diseases for individuals. In 2019, the Chinese government spent 845.916 billion yuan on medical insurance, accounting for 46.95% of the total health expenditure [7]. By the end of 2020, 1.361 million people in China had obtained full coverage of basic medical insurance, with coverage stabilizing at over 95% [8]. Despite these efforts, China’s coverage remains insufficient [9]. Xu et al. reported that the additional annual expenditure on inpatient care (per member) in elderly chronic disease families (ECD-family) was 37–45% more than the annual spending in the control group. Moreover, approximately 35–43% of the average ECD-family’s expenditure was spent on out-of-pocket inpatient and outpatient care [10]. Therefore, under the current health insurance policy, the economic burden of disease in the ECD-family is much heavier than in families without chronic diseases. In summary, the universal health care system should be enhanced to target vulnerable groups and to help families trapped in the vicious cycle of poverty and disease.

Additionally, various factors influence the occurrence of catastrophic health expenditures (CHE). First, socio-demographic characteristics are the main determinants for the occurrence of CHE. Xu et al. reported that factors such as age, family size, and income significantly impact the health expenditure of urban Chinese households [11]. Another study showed that households headed by females and middle-aged adults (40–59 years) are less likely to suffer CHE. However, the likelihood of CHE is higher in households with low economic levels and elderly family members [12]. In addition, householders’ education level, family’s residential area, economic status, living conditions, and having disabled household members were also associated with CHE incidence [12–16]. Second, health services demand and utilization are also closely related to CHE incidence [17–20]. Previous literature suggests that improving the health status of residents and reducing inpatient services utilization could reduce the incidence of CHE in families [17]. More health care utilization and the average number of illness episodes among household members could increase CHE incidence [18]. Third, social security equity factors are a significant aspect in the probability of incurring CHE. Specifically, health insurance coverage, equality financing, and community-based health insurance schemes have been established as having a protective function against the economic burden of disease [21–24]. Moreover, maintaining the high population coverage and deepening the cost coverage of the medical insurance schemes is considered an effective institutional tool to reduce CHE incidence. Pandey et al. suggested that public policy should focus more on vulnerable populations with high CHE incidence by expanding health insurance coverage and providing high-quality health services for them [24]. Based on the above theoretical framework analysis, we selected variables from the three dimensions of sociodemographic characteristics, health service demand and utilization, and social security equity to establish the determinants for CHE occurrence in chronic disease families.

## Methods

### Study design

The data used in this study were obtained from the 2018 China Health and Retirement Longitudinal Study (CHARLS) database. It is the most nationally representative study of middle-aged and older adults in China and is coordinated with leading international studies in the Health and Retirement Research (HRS) model [25]. The 2018 CHARLS database used in this study covers 150 regions, 450 villages/urban communities, and 17,708 people in 10,257 families, reflecting the overall situation of China’s middle-aged and older adult population. It comprehensively analyses the living conditions of middle-aged and older adults aged over 45 years regarding seven aspects: personal information, family members, health status, medical insurance status, income, expenditure, and assets status.

### Data extraction

Since CHE constitutes household-level data, the data in this study were based on households. The related variables involved in calculating CHE and the variables of medical services comprised the core variables of the study. The data cleaning process and the raw data collection were conducted as follows. Family food expenditure was determined by the question, “In the past week, how much did your household spend on food?” Missing cases were deleted because they could not be supplemented. Household out-of-pocket medical expenses were determined by the question, “In the last year, how much did your household spend on direct and indirect medical expenses?” Missing values for out-of-pocket household medical expenses were supplemented by calculating the sum of out-of-pocket outpatient and inpatient expenses. Household size was determined by the question, “Of all people listed here, which are your household members?” Missing values were supplemented by the number of persons in the database under the same family code. After excluding missing and invalid values that could not be supplemented, 9186 households were obtained from the database, accounting for 10% of missing data.

**Fig. 1:**
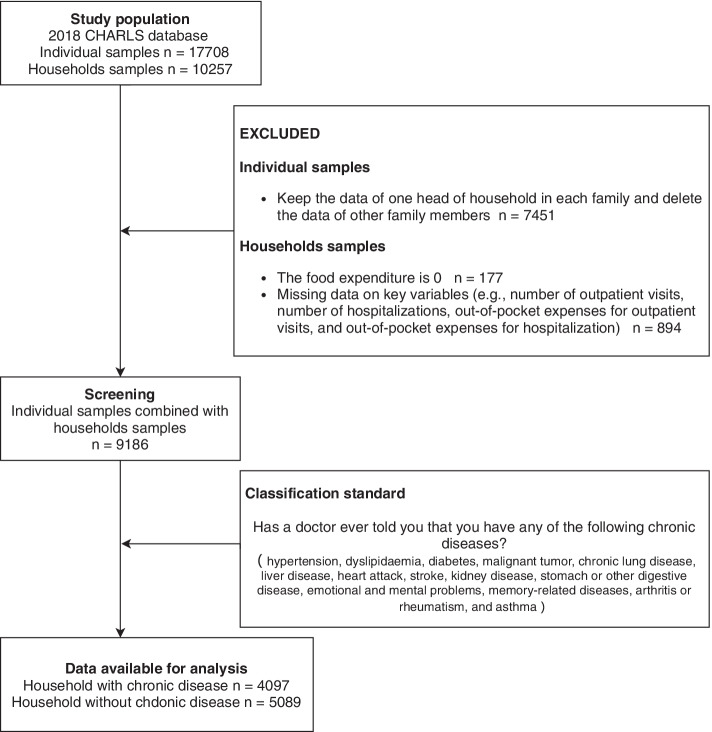
Sample screening flow chart

In addition, we used the question “Has a doctor ever told you that you have any of the following chronic diseases?” as the standard to select the chronic disease sample populations. This question contains 14 sub-questions, including hypertension, dyslipidaemia, diabetes, malignant tumour, chronic lung disease, liver disease, heart attack, stroke, kidney disease, stomach or other digestive diseases, emotional and mental problems, memory-related diseases, arthritis or rheumatism, and asthma. Individuals were considered ill if they answered “yes” to any of the 14 sub-questions and were sick, and not ill if they answered “no” to all 14 questions. According to family ID, individuals with chronic diseases were summarized, resulting in 4097 households with chronic disease members. Finally, we found that the critical variables in calculating CHE or regression models were not missing. The logical verification was accurate; therefore, no further exclusion of families from the database was conducted. The specific sample screening process is shown in Fig. 1.

### Variable selection

#### Calculation of catastrophic health expenditures

We took the CHE occurrence as the dependent variable, and this study adopted the method recommended by WHO to calculate CHE [26]. All expenditure-related variables must first be converted to monthly data based on the ordinary month’s consumer price index (CPI). The key variables are as follows: out-of-pocket health expenditure (OOP), household consumption expenditure (*exp*_*h*_), household capacity to pay (*ctp*_*h*_), food expenditure (*food*_*h*_), and household size.

First, we calculated the share of food expenditure per household (*foodexp*_*h*_) by dividing the household’s food expenditure by the household consumption expenditure. Second, we used a household equivalence scale instead of actual household size for household consumption size. The calculation is as follows:

$${eqsize}_h={hhsize}_h^{\beta }$$

*hhsize*_*h*_ is the actual household size surveyed in the CHARLS database and the value of the parameter β. This is estimated from household surveys in 59 countries and is 0.56.

Third, based on the equivalent household size, the equivalent food expenditure (*eqfood*_*h*_) can be obtained by dividing household’s food expenditure by the *eqsize*_*h*_. Then the poverty line (*pl*) and subsistence expenditure (*se*_*h*_) can also be calculated based on *eqfood*_*h*_. To reduce the measurement error, we used *foodexp*_*h*_ between the 45th (*food*45) and 55th (*food*55) place in *exp*_*h*_.

$${eqfood}_h=\frac{food_h}{eqsize_h}$$

$$pl=\frac{\sum {w}_h\ast {eqfood}_h}{\sum {w}_h}$$

where *food*45 < *foodexp*_*h*_ < *food*55

$${se}_h= pl\ast {eqsize}_h$$

Fourth, household’s capacity to pay (*ctp*_*h*_) is defined as household non-subsistence spending. According to whether food subsidies, coupons, self-produced food, and other non-cash consumption are considered, the measurement methods can be divided into the following:

$${ctp}_h={\mathit{\exp}}_h-{se}_h\ \mathrm{if}\ {se}_h\le {food}_h$$

$${ctp}_h={\mathit{\exp}}_h-{food}_h\ \mathrm{if}\ {se}_h\ge {food}_h$$

Fifth, out-of-pocket health payments share of household capacity to pay (*oopctp*_*h*_), which refers to the family medical cost burden, is defined as the percentage of out-of-pocket payments of the household as a percentage of *ctp*_*h*_.

$${ oop ctp}_h=\frac{oop_h}{ctp_h}$$

Finally, based on the calculations of the above variables, CHE occurs when *oopctp*_*h*_ equals or exceeds 40%, as follows:

$${cata}_h=1\ \mathrm{if}\ {oopctp}_h\ge 0.4$$

*cata*_*h*_ = 0 if *oopctp*_*h*_ < 0.4

#### Independent variables

Explanatory variables included the following: (1) sociodemographic factors, including the householder’s age, sex, marital status, education level, and the family’s main residence (urban or rural), region, economic level (ranked according to the per capita household expenditure of the sample households and divided into five equal groups), household size, and whether the householder participates in the labour; (2) health services demand and utilisation factors, including the householder’s self-rated health, whether the householder has been an outpatient in the past month and whether the householder used inpatient service in the past year, whether households contained disabled members, or whether there is an older adult aged 65 years or above in the household; (3) social security equity factors, namely, the participation in medical insurance status of the householder, including urban employee basic medical insurance (UEBMI), urban resident basic medical insurance (URBMI), new rural cooperative medical schemes (NRCMS), urban and rural residents basic medical insurance (URRBMI), and other health insurance, or uninsured. Specific variable assignments are shown in Table 1.

**Table 1:** Selection and description of variables

Variable	Description
Dependent variable	
Catastrophic health expenditures (CHE)	0 = The household did not occur catastrophic health expenditures; 1 = The household occurred catastrophic health expenditures
Independent variables	
Sociodemographic factors	
Sex of householder	0 = Female; 1 = Male
Age of householder	Numerical variable
Marital status of householder	0 = Single (including divorced, single, widowed and unmarried); 1 = Married
Family’s main residence	0 = Rural; 1 = Urban
Educational level of householder	1 = Illiterate; 2 = Primary school; 3 = Junior high school and above
Household size	1 = Single-person households; 2 = Two-person households; 3 = Cohabitation of three or more persons
Region	1 = East; 2 = Central; 3 = West
Family economic level	1 = The poorest level; 2 = Sub-poverty level; 3 = General level; 4 = Wealthier level; 5 = The wealthiest level
Whether the householder participates in the labour	0 = No; 1 = Yes
Health services demand and utilisation factors	
Householder self-rated health	0 = Good; 1 = Bad
Householder used outpatient service in the past month	0 = No; 1 = Yes
Householder used inpatient service in the past year	0 = No; 1 = Yes
Household with disabled members	0 = No; 1 = Yes
Household with members aged over 65 years	0 = No; 1 = Yes
Social security equity factors	
Householder participated in medical insurance type	0 = URRBMI; 1 = UEBMI; 2 = NRCMS; 3 = URBMI; 4 = Other medical insurance (including uninsured)
Disease factors	
Type of chronic disease	1 = 1 type (The householder suffers from a chronic disease); 2 = >1 type (The householder suffers from more than one chronic disease)
Heart disease	0 = The householder did not suffer from the disease; 1 = The householder suffers from the disease
Stroke	0 = The householder did not suffer from the disease; 1 = The householder suffers from the disease
Malignant tumour	0 = The householder did not suffer from the disease; 1 = The householder suffers from the disease
Chronic lung disease	0 = The householder did not suffer from the disease; 1 = The householder suffers from the disease
Diabetes	0 = The householder did not suffer from the disease; 1 = The householder suffers from the disease

### Statistical analysis

#### Logistic regression model

Logistic regression is a type of general linear model, that is, regression analysis with dichotomous variables (0 and 1) as the response variables and the probability of taking a specific value as the model output. It is mainly used to predict the likelihood or screen the risk factors of an event. For the binary response variable *y*, the linear logistic regression model has the following form [27–29]:

$$logit\Big\{\left({y}_i={y}_1|{X}_i\right\}= logit\left({p}_i\right)=\log \left[\frac{p_i}{1-{p}_i}\right]={\beta}_0+{\beta}_1{X}_1+\cdots +{\beta}_j{X}_j+\cdots +{\beta}_k{X}_k={\beta}_0+{\beta}^{\prime }{X}_i$$

where *p*_*i*_ = *probability* (*y*_*i*_ = *y*_1_ ∣ *X*_*i*_}, *β*_0_ is the *y* intercept, *β*^′^ is the vector of slope parameters, *y*_*i*_ is the first ordered level of *y*, and *X*_*i*_ is the vector of explanatory variables.

In the above logistic regression equation, the response variable is the logarithmic odds ratio (log) of *y* = 1. The regression coefficient is defined when other predictive variables are held constant, the change in the logarithmic odds ratio of the response variable that can be induced by a change in one unit of the predictive variable. Therefore, we used binary logistic regression to understand the association between dependent and independent variables in the model and calculated 95% confidence intervals (CI) and odds ratio (OR). In addition, we conducted a multicollinearity diagnosis for the 15 independent variables in the regression model. The results showed that all variables’ variance inflation factor (VIF) values were between 1 and 2 and did not reach 5; thus, there was no multicollinearity problem among the independent variables. Data were analysed using IBM SPSS 26.0.

## Results

### Sample characteristics

The descriptive statistics results are summarized in Table 2. A total of 4097 families in the sample had a member with diseases. Statistical analysis revealed a significant association between CHE incidence and the age of householder, family’s main residence, education level of householder, household size, and if householder participated in medical insurance type. Moreover, the incidence of CHE in households with householders aged 65 years or above (30.24%) was significantly higher than those with householders aged below 65 years (17.89%). In rural households, the incidence of CHE was higher than that in urban households, with a gap of 8.80 percentage points (pp). With the improvement in the educational level of householders, the incidence of CHE was significantly decreased by 14.43 pp. In addition, people who lived alone experienced CHE more than family members living together, with an increased probability of up to 10.73 pp. Comparing several types of health insurance coverage, the incidence of CHE was highest (27.46%) when the householder participated in the URRBMI.

**Table 2:** Characteristics of the sample

Variable		Middle-aged and older adults’ families with chronic disease		
		Frequency	Proportion(%)	Incidence of CHE(%)
Sex of householder	Male	1864	45.50	23.07
	Female	2233	54.50	23.96
Age of householder	< 65	2219	54.16	17.89^**^
	≥65	1878	45.84	30.24
Family’s main residence	Urban	1240	30.27	17.42^**^
	Rural	2857	69.73	26.22
Educational level of householder	Illiterate	978	23.87	30.67^**^
	Primary school	1783	43.52	25.18
	Junior high school and above	1336	32.61	16.24
Marital status of householder	Married	3199	78.08	23.94
	Single (including divorced, single, widowed and unmarried)	898	21.92	22.16
Household size	Single-person households	522	12.74	27.01^**^
	Two-person households	2930	71.52	24.54
	Cohabitation of three or more persons	645	15.74	16.28
Region	East	1309	31.95	25.44
	Central	1380	33.68	22.32
	West	1408	34.37	23.01
Householder participated in medical insurance type	URRBMI	477	11.64	27.46^**^
	UEBMI	623	15.21	17.66
	NRCMS	2602	63.51	25.37
	URBMI	192	4.69	17.19
	Other medical insurance (including uninsured)	203	4.95	15.27

^*^ *P* < 0.05, ^**^ *P* < 0.01

### CHE incidence in the whole population versus families of the middle-aged and older adult population regarding socioeconomic factors

**Fig. 2:**
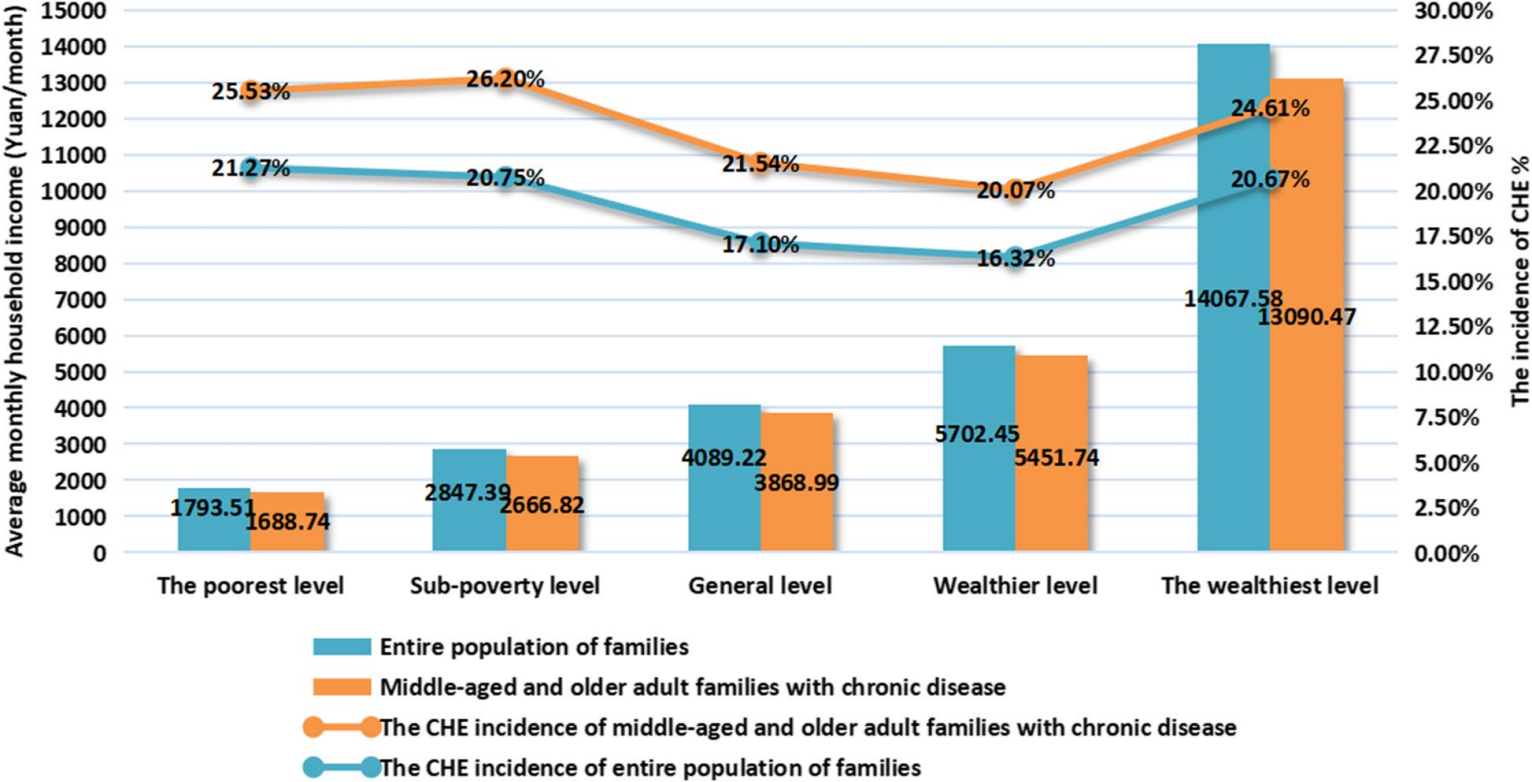
The mean comparison of monthly household income of different groups in different economic groups

This study calculated and compared the average monthly income and CHE incidence of families with and without disease in different economic groups, as shown in Fig. 2.

Statistical analysis revealed a significant association between the mean monthly income of middle-aged and older adult families with chronic disease (*P* < 0.05). As shown in Fig. 2, the mean monthly income of the entire population of families was significantly higher than that of middle-aged and older adult families with chronic disease, especially at the wealthiest level, reaching 1.07 times. Additionally, the CHE incidence difference between middle-aged and older adult families with chronic disease and the entire population in each economic group also showed a significant association (*P* < 0.05). The overall position of the line chart in Fig. 2 illustrates that, in all economic groups, the incidence of CHE in middle-aged and older adult families with chronic diseases was significantly higher than that in the entire population. Among all economic groups, the gap in CHE incidence was the largest in the sub-poverty group by 5.45 pp., and between middle-aged and older adult families with disease and the entire population. CHE incidence in the middle-aged and older adult families with chronic disease was ranked from high to low as sub-poverty (26.20%), poorest (25.53%), wealthiest (24.61%), general (21.54%), and wealthier level families (20.07%). However, the CHE incidence of the entire population of families was highest in the poorest level families (21.27%), and lowest in the wealthier level families (16.32%).

### The CHE incidence of different health service demand and utilisation

Inpatients and outpatients with chronic diseases were taken as the research subjects to compare CHE incidence in different chronic diseases (Table 3). We also analysed the populations with the highest incidence of CHE under various health services to identify vulnerable targets for current health insurance schemes.

**Table 3:** The incidence of CHE and frequency in households with different diseases under different health service demand and utilisation conditions

Variable		Families without disease		Families with disease											
				Total		Heart disease		Stroke		Malignant tumour		Chronic lung disease		Diabetes	
		Frequency (N)	Incidence of CHE	Frequency (N)	Incidence of CHE	Frequency (N)	Incidence of CHE	Frequency (N)	Incidence of CHE	Frequency (N)	Incidence of CHE	Frequency (N)	Incidence of CHE	Frequency (N)	Incidence of CHE
Householder self-rated health	Good	4277	13.65%^##^	2584	17.53%	367	17.71%^**^	192	23.44%^**^	56	28.57%^**^	256	21.88%^**^	283	18.02%^**^
	Bad	812	27.09%^##^	1513	33.84%	299	36.79%	256	42.58%	67	55.22%	237	36.71%	186	37.10%
Household with members aged over 65 years	Yes	2015	22.28%^##^	2049	29.97%	370	29.73%^*^	299	38.80%^**^	71	50.70%	254	33.07%^*^	245	31.02%^**^
	No	3074	11.55%^##^	2048	17.14%	296	21.96%	149	25.50%	52	32.69%	239	24.69%	224	19.64%
Household with disabled members	Yes	806	23.20%^##^	1001	29.47%	182	29.12%	159	40.25%	39	48.72%	149	34.23%	124	32.26%
	No	4283	14.41%^##^	3096	21.64%	484	25.21%	289	31.14%	84	40.48%	344	26.74%	345	23.19%
Type of chronic disease	1 type	–	–	2519	21.91%	256	24.22%	169	31.36%	44	36.36%	170	27.06%	156	22.44%
	≥1 type	–	–	1578	26.17%	410	27.56%	279	36.20%	79	46.84%	323	30.03%	313	27.16%
Householder used outpatient service in the past month	Yes	662	28.10%^##^	894	35.91%	155	40.65%^**^	107	47.66%^**^	30	53.33%	160	41.25%^**^	104	38.46%^**^
	No	4427	13.96%^##^	3203	20.11%	511	21.92%	341	30.21%	93	39.78%	333	23.12%	365	21.92%
Householder used inpatient service in the past year	Yes	540	34.81%^#^	1006	41.25%	199	36.18%^**^	203	46.31%^**^	62	66.13%^**^	168	44.64%^**^	125	35.20%^**^
	No	4549	13.54%^##^	3091	17.79%	467	22.06%	245	24.49%	61	19.67%	325	20.92%	344	22.09%

^*^ *p* < 0.05, ^**^ *p* < 0.01. Comparisons were made between groups for different conditions under each disease

^#^ *p* < 0.05, ^##^ *p* < 0.01. Comparisons were made between groups of families without diseases and families with diseases under different conditions

We first stratified the sample families according to the five major chronic diseases of concern in this study. Then, we compared the incidence of CHE in families under different health service demands and use conditions. The results showed a significant association in CHE incidence between the chronic disease families and those without diseases (*P* < 0.05). The CHE incidence of families without diseases was significantly lower than that in families with chronic diseases in health service utilization conditions. Specifically, when the householder had been an outpatient in the past month, the difference in CHE incidence between families with chronic diseases and that without diseases was the highest, and the maximum gap reached up to 7.81 pp. Moreover, when the householder’s self-rated health status was “good”, the contrast of CHE incidence between families with chronic disease and families without chronic disease was the most minor (3.88 pp).

Specifically, self-reported health status and inpatient utilization over the past year were significant variables for the occurrence of CHE (*P* < 0.01). The highest CHE rate was 66.13% in householders with a malignant tumour who had used hospital services in the past year. Similarly, householders with chronic lung disease who received inpatient services in the past year had a higher incidence of CHE (44.64%) than did other persons with this disease. Except for malignant tumours, CHE incidence of the other four diseases showed significant differences when a family member was over 65 years old and there had been outpatient use in the past month (*P* < 0.05). On this basis, the incidence of CHE was highest when householders had a stroke and had used outpatient services in the past month (47.66%). In addition, householders with heart disease (40.65%) or diabetes (38.46%) also had the highest incidence of CHE when they had used outpatient services in the past month versus those with other conditions.

### The CHE incidence of householders with different diseases under different insurance types

**Fig. 3:**
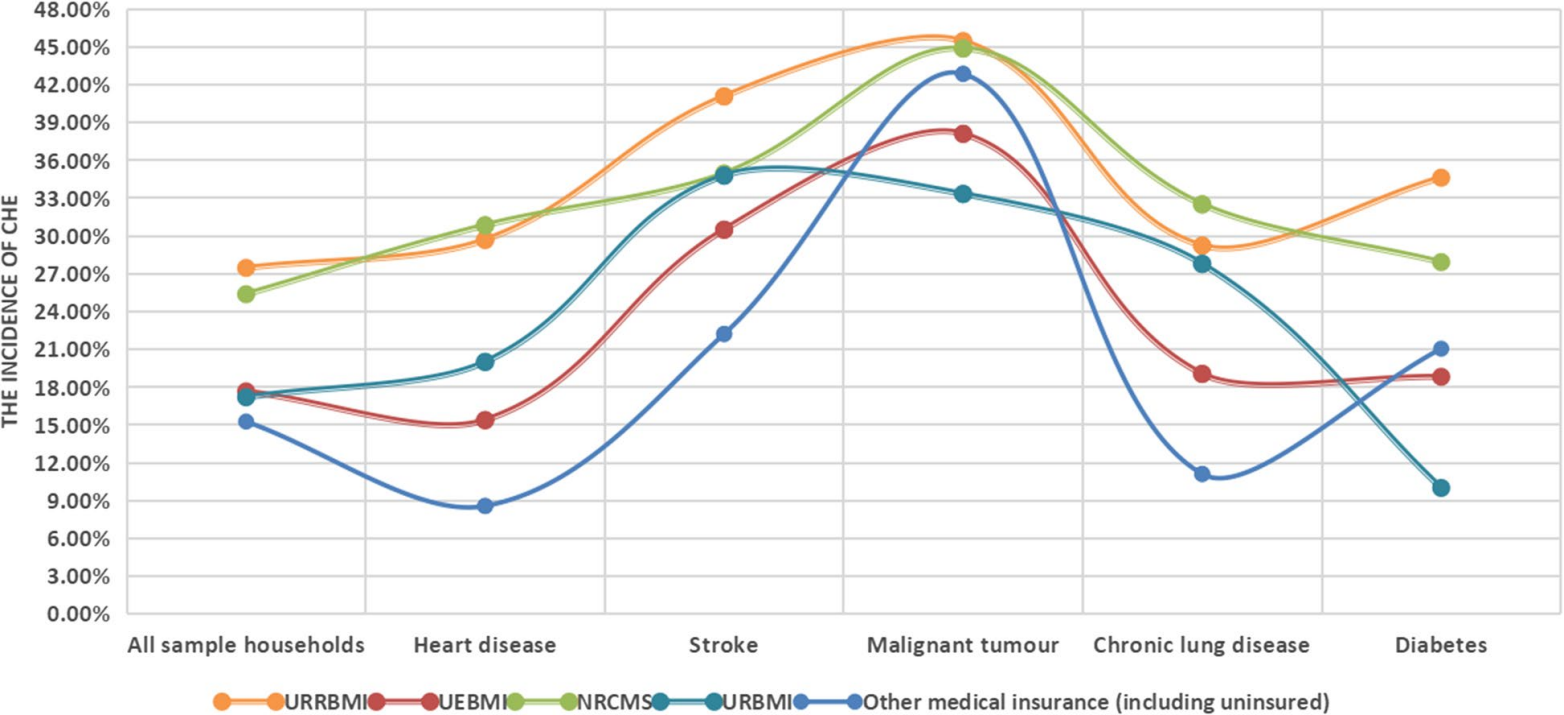
Comparison of the incidence of CHE among people with the different chronic diseases under different types of medical insurance

We compared the incidence of CHE when household heads participated in different types of medical insurance (Fig. 3). The incidence of CHE was the lowest (15.27%) when the householder participated in other medical insurance (including uninsured) and was the highest (27.46%) when the householder participated in the URRBMI (*P* < 0.05). Classifying the families by disease, in householders with heart disease (30.84%) or chronic lung disease (32.50%), the incidence of CHE was the highest in the NRCMS group (*P* < 0.05). Regarding householders with stroke, the incidence of CHE was significantly higher in the URRBMI group (41.07%) than in the other medical insurance (including uninsured) groups (22.22%) (*P* < 0.05). Additionally, the incidence of CHE in the URBMI group (33.33%) was significantly lower than that in the URRBMI group (45.45%) when householders had a malignant tumour (*P* < 0.05).

### Regression analysis of influencing factors of CHE incidence in diseased families

In Table 4, the Hosmer & Lemeshow test were used to judge the model fit effect, *x*^2^(8) = 2.85, *P* = 0.94, which showed that the model had a high goodness of fit.

**Table 4:** Results of a logistic regression model

Variable (reference)	OR	95% CI of OR	***p***-value
Age of householder	1.01	0.10–1.02	0.15
Region (East)	Ref		
Central	0.77	0.63–0.93	0.00^**^
West	0.78	0.65–0.95	0.01^*^
Family economic level (The poorest level)	Ref		
Sub-poverty level	0.10	0.78–1.27	0.97
General level	0.83	0.65–1.07	0.15
Wealthier level	0.69	0.53–0.89	0.00^**^
The wealthiest level	0.87	0.67–1.12	0.27
Household size (Single-person households)	Ref		
Two-person households	1.08	0.85–1.36	0.53
Cohabitation of three or more persons	0.70	0.51–0.95	0.02^*^
Whether the householder participates in the labour (No)	Ref		
Yes	0.82	0.69–0.98	0.03^*^
Household with members aged over 65 years (No)	Ref		
Yes	1.43	1.11–1.84	0.00^**^
Households with disabled members (No)	Ref		
Yes	1.28	1.07–1.52	0.00^**^
Householder used outpatient service in the past month (No)	Ref		
Yes	2.16	1.81–2.57	0.00^**^
Householder used inpatient service in the past year (No)	Ref		
Yes	2.73	2.30–3.24	0.00^**^
Whether the householder suffers from heart disease (No)	Ref		
Yes	1.12	0.92–1.38	0.27
Whether the householder suffers from stroke (No)	Ref		
Yes	1.32	1.04–1.66	0.02^*^
Whether the householder suffers from malignant tumour (No)	Ref		
Yes	2.02	1.35–3.00	0.00^**^
Whether the householder suffers from chronic lung disease (No)	Ref		
Yes	1.14	0.91–1.43	0.27
Whether the householder suffers from diabetes (No)	Ref		
Yes	1.13	0.89–1.43	0.31
Householder participated in medical insurance type (URRBMI)	Ref		
UEBMI	0.49	0.36–0.67	0.00^**^
NRCMS	0.92	0.73–1.16	0.47
URBMI	0.56	0.36–0.89	0.01^*^
Other medical insurance (including uninsured)	0.45	0.28–0.70	0.00^**^
_cons	0.14		0.00
Model validation	Chi-square		p-value
Hosmer & Lemeshow test	2.85		0.94

Note: ^*^ *P* < 0.05, ^**^ *P* < 0.01. Sample size: 4097

On one hand, according to the logistic regression results in Table 4, we found a higher incidence of CHE in the following conditions. Households with inpatient members had 2.73 times (95% CI 2.30–3.24) more probability of suffering CHE than those with no inpatient members. Moreover, the likelihood of CHE was 2.16 times (95% CI 1.81–2.57) higher for those who had used outpatient services in the past month than those who had not. The probability of incurring CHE was 2.02 times (95% CI 1.35–3.00) more in householders with a malignant tumour than in householders without. Households with members aged 65 years and older were 1.43 times more likely (95% CI 1.11–1.84) to experience CHE than households without older adult members. Households with members who had a disability had 1.28 times (95% CI 1.07–1.52) higher odds of facing CHE than those without. In addition, householders who had had a stroke were 1.32 times more likely (95% CI 1.04–1.66) to face CHE than those in the reference group.

On the other hand, the information provided in Table 4 shows that the incidence of CHE was lower in the following cases. Regarding medical insurance, except for NRCMS, the other three types had lower CHE incidence than URRBMI. When the householder participated in the UEBMI, they were 0.49 times less likely (95% CI 0.36–0.67) to face CHE than URRBMI. If the householder participated in the URBMI, that household was 0.56 times less likely (95% CI 0.36–0.89) to face CHE than householders who participated in the URRBMI. Householders with other medical insurance (including uninsured) were 0.45 times less likely (95% CI 0.28–0.70) to experience CHE than householders covered by URRBMI. Regarding different family sizes, the incidence of CHE was 0.70 times (95% CI 0.51–0.95) lower in households with three or more cohabitants than in single-person households. Furthermore, among the five levels of family economic groups, the wealthiest household had 0.69 (95% CI 0.53–0.89) lower odds of CHE compared to the poorest household. Finally, households living in central regions were 0.77 times less likely (95% CI 0.63–0.93) to experience CHE than those living in the east. Households living in the western region were also 0.78 times less likely (95% CI 0.65–0.95) to face CHE than those living in the eastern region.

## Discussion

### The relative affluence of the middle-aged and older adult population does not significantly reduce the family economic burden of disease

The health characteristics of middle-aged and older adult groups mainly include two aspects. First, older adults have high utilisation of health services; however, due to their vulnerable social and economic capacity, their unmet health service needs are also high. Second, middle-aged and older adults with diseases experience the onset of a slow process, and develop complications or organ failure. Under the dual support of physical and social vulnerability, the middle-aged and older adult population have always been a vulnerable population that has attracted the attention of health service providers. The 2018 survey results showed that the two-week consultation and annual hospitalisation rates of Chinese people aged 65 years and above were 42.6 and 27.2%, respectively, which are far higher than those of other age groups [7]. Older adults consume more medical resources than permanent residents. The utilization rate of outpatient services will increase with age, and medical expenses will gradually increase [30].

.Our results show that for middle-aged and older adult families with chronic disease, the incidence of CHE in the wealthiest level group was 24.61%, which was not the group with the lowest incidence of CHE as expected. Additionally, the incidence of CHE in the poorest affected families was not the highest (25.53%), which is consistent with Li et al. [17] This indicates that for middle-aged and older adult families with chronic diseases, relatively affluent economic status has few advantages against disease burden.

The above conclusion is not only applicable to families at different economic levels, but also to regions with varying economic levels. For example, the logistic regression results in Table 4 show that the likelihood of CHE in central and western regions was lower than that in the eastern areas. In this context, the eastern region is a coastal region, and its economic level is much higher than that of the central and western regions. In 2019, the GDP of the eastern, central, and western regions was 510 million yuan, 220 million yuan and 210 million yuan, respectively [31]. Also in 2019, 84.25 million people were hospitalized in the eastern region, 1.29 times that of the central region, and 1.35 times that of the western region [7]. Based on the statistical data, we infer that the likelihood of CHE is higher in the eastern region due to the higher utilization of health services. For the middle-aged and older adult population with chronic diseases, the impact of economic level on CHE is not dominant; however, the utilization of health services is. That is, even for the relatively affluent elderly, chronic diseases significantly raise the probability of incurring CHE after using health services. Regarding the results of this study, some scholars have highlighted that another reason for this situation is that low-income groups lack the necessary economic conditions to access health services. Hence, the utilisation rate of health care is relatively low [17, 32, 33].

### .Although the medical security for middle-aged and older adults with chronic diseases has progressed, it has not been accurately implemented

In China, it is estimated that more than 300 million urban and rural residents experience the two predominant diseases, hypertension and diabetes. To reduce the burden of outpatient medication for urban and rural residents with hypertension and diabetes, the 2019 Government Work Report proposed that ‘medicines for hypertension and diabetes should be covered by medical insurance’. Based on hospitalisation, more than 50% of patient expenses for drugs to lower blood pressure and blood sugar in the outpatient clinic were paid within the policy scope [34, 35]. This shows that China’s current medical insurance policy has begun to attend to the medication burden for chronic diseases. However, the current medical insurance policy has a ‘one-size-fits-all’ approach for the whole population and does not identify vulnerable groups. Studies demonstrate that this approach to designing health insurance results in unequal benefits for different people [32]. For example, surveys show that the per capita medical expenses for diabetes patients aged 65 years and above were 2551.97 yuan, which is higher than the average for the whole population [36]. Moreover, our findings are consistent with Sayem Ahmed et al., who reported that CHE are higher when an older person is in the household [37]. Although China’s current medical insurance policy has focused on the cost of outpatient drug that are urgently needed by the sick population, it has not focused on vulnerable people. Designing differentiated reimbursement schemes according to different vulnerability characteristics should be prioritised to more deeply reform the medical insurance system.

In the sample population of this study, we found that CHE had the highest incidence in URRBMI (Fig. 3). The URRBMI is a medical insurance system that integrates the URBMI and NRCMS proposed by the State Council in 2016 [38]. Presently, many provinces still use the current financing mechanism of the NRCMS and the URBMI when integrating the URRBMI, leading to a low financing scale [39]. Additionally, some scholars highlight that the average number of medical visits, outpatient expenses, and hospitalisation expenses of rural residents are lower than those of urban residents because of lower availability of medical services. Therefore, high-income groups can consume more medical resources, leading to the reverse redistribution phenomenon of rural residents supporting urban residents [40]. Therefore, the URRBMI eliminates the differences based on status and household registration. Still, it cannot eliminate the different forms of financing and treatment within the system, leading to a low-security level [41]. Interestingly, we found that CHE incidence for people under other medical insurance (including the uninsured) was the lowest. This finding is consistent with that of Wang et al. [42] This result is because when individuals are insured, they are more likely to use medical services to improve their health [43]. Thus, when householders participate in medical insurance, the frequency of use of outpatient and inpatient services increases significantly, leading to a higher economic burden of disease [44].

.Regarding specific diseases, Fig. 3 shows that malignant tumours had a high incidence of CHE under the five insurance types. Table 4 shows that the likelihood of CHE was 2.02 times higher for householders with malignant tumours than those without. In 2019, the number of deaths due to malignant tumours accounted for 25.73% of China’s total deaths. The per capita inpatient medical expenses reached up to 29,737.8 yuan, which was much higher than other diseases in the same year [7]. Liu et al.’s study showed that drug costs are the main burden of hospitalisation costs for malignant tumours [45]. China’s health security authorities have successfully included more than 40 types of anti-cancer drugs in the B category of medical insurance through three rounds of medical insurance drug negotiations to reduce the drug burden of cancer patients [46]. According to the monitoring results of the delivery of negotiated drugs by the Medical Insurance Bureau, the actual reimbursement rate of negotiated drugs is more than 60% [47]. For example, according to information from the State Medical Products Administration, the price of domestic imatinib (Xinwei) is only one-ninth of the price of imported imatinib (Gleevech) (more than 10,000 yuan). However, the current medical insurance drug list still reimburses this at the same rate. In 2019, Chinese residents’ annual per capita disposable income was 30,732.8 yuan. According to the standard of two cartons per month, there remains a large gap between the OOP expenses of imported drugs and domestic drugs after reimbursement, which is burdensome. In conclusion, due to the heavy drug burden and low reimbursement level of medical insurance, the family disease burden of patients with malignant tumours is heavy, leading to a high incidence of CHE. This suggests that China’s current medical insurance policy should expand the coverage of cancer drug use and distinguish the reimbursement of imported drugs from domestic drugs. Nonetheless, it is gratifying to see considerable progress in the latest medical insurance drug reimbursement adjustment in 2022.

### The utilisation of outpatient services is also an important factor affecting the occurrence of CHE

Our study found that inpatient and outpatient services were the two most critical factors that increased the likelihood of CHE. In recent years, China’s medical insurance reform has begun to expand the reimbursement ratio of inpatient expenses; however, outpatient expenses reimbursement remains insufficient [9, 48]. Meanwhile, the average medical cost of outpatient visits for Chinese residents increased from 166.8 yuan in 2010 to 290.8 yuan in 2019 [7]. This indicates that the burden of the outpatient expense of Chinese residents is increasing. Furthermore, existing studies have shown that outpatient burden is critical, especially for middle-aged and older adults with chronic diseases. A study of household health costs in Vietnam demonstrated that the long-term cumulative burden of outpatient care even exceeded the burden of hospitalization [49]. In addition, Goeppel et al. focused more on outpatient services when assessing UHC for middle-aged and older adults with chronic diseases in several countries [50]. Xiaoyun Liu et al. also pointed out that due to the low income of older adults with chronic diseases, although outpatient fees are mostly low, they still incur CHE [51]. This suggests that the outpatient service utilisation rate of the older adult population was high, and the cost burden was heavy.

In recent years, to reduce the burden on residents, China’s current medical insurance policy focused more on reimbursing inpatient expenses for the insured. In 2019, the pooling fund for inpatient medical expenses covered by China’s medical insurance policy reached approximately 70%, while the reimbursement rate for outpatient medical expenditures was only approximately 50%. Furthermore, Chaofan Li mentioned in their study that when a patient has a second inpatient visit within a year, the inpatient visit deductible is eliminated. This further reduces the cost burden of multiple hospitalizations within a year [52]. Regarding outpatient expenses, patients need to pay higher out-of-pocket expenses when using outpatient services because of their lower reimbursement rate and upper reimbursement limit [48, 53, 54]. Therefore, while reducing the burden of hospitalisation for residents, medical insurance policies should also consider the reimbursement of outpatient expenses. This is especially true for vulnerable groups, such as chronic disease patients and middle-aged and older adults.

### Focusing on the vulnerability characteristics of populations with high probability of CHE

**Fig. 4:**
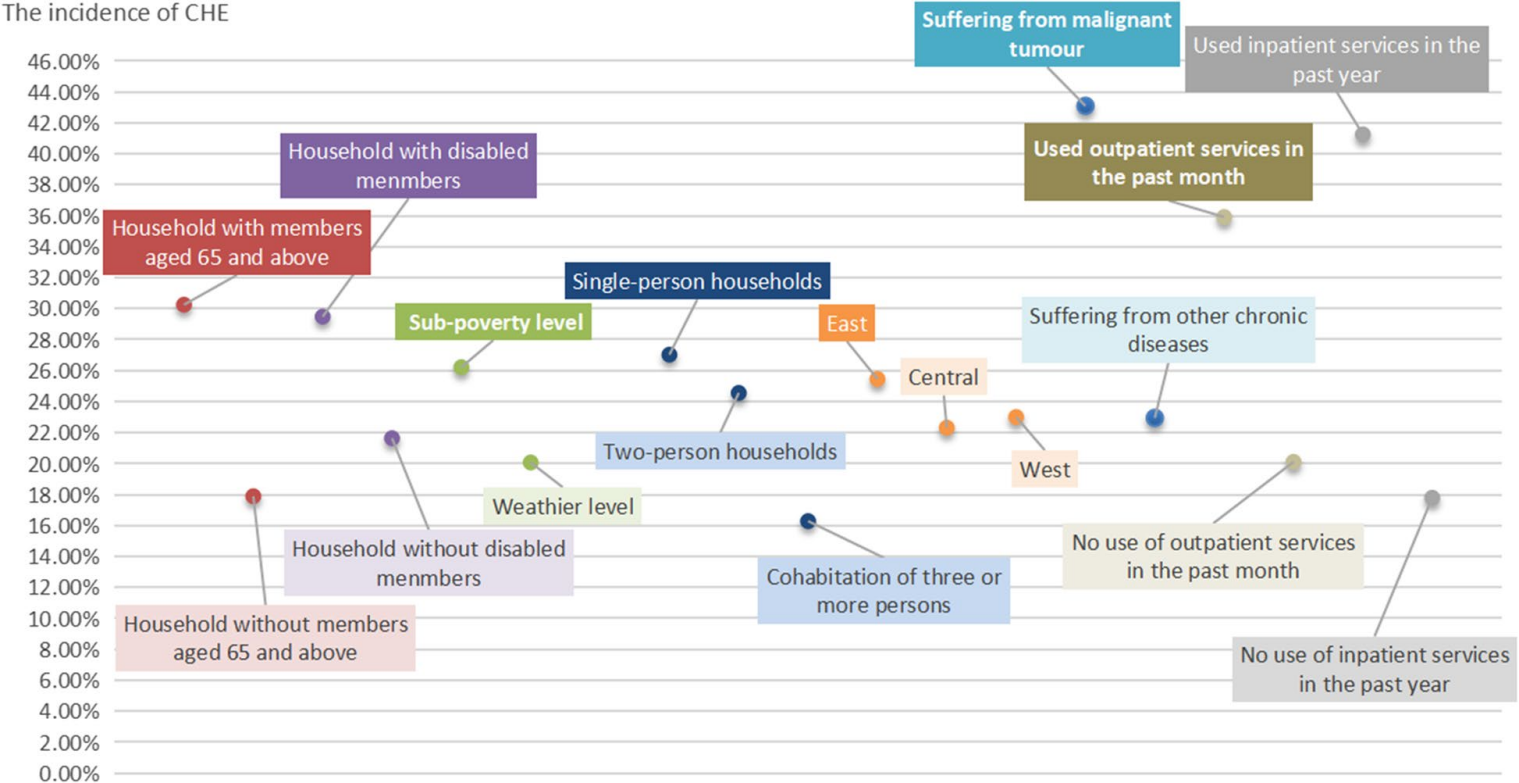
Summary of vulnerability characteristics of populations with a high probability of incurring CHE

This study focused on middle-aged and older adults with chronic diseases. Therefore, the vulnerability characteristics of populations with a high probability of CHE can be initially identified and summarised. Regarding sociodemographic characteristics, a household with a member who is disabled or an older adult aged over 65 years is more likely to suffer CHE, which increases the burden of health care on the family. Compared with households at other economic levels, the sub-poverty group had the highest probability of incurring CHE, while the wealthier level had the lowest probability. Additionally, middle-aged and older adults who live alone and lack the help of family members are more likely to face CHE. Furthermore, the eastern region has a developed economy, a high utilisation rate of health services, and a heavy burden of medical expenses. Moreover, middle-aged and older adults with malignant tumours have a higher likelihood of CHE than those suffering from other chronic diseases. Finally, middle-aged and older adults with chronic diseases have a high utilization rate of health services. In addition to high hospitalization costs, a high frequency of outpatient service utilization will also lead to a high medical burden. Figure 4 presents a visual summary of groups with high CHE incidence as discussed above.

Due to the diversity of population vulnerability characteristics, China’s medical insurance system must focus on several aspects. Middle-aged and older adults with many diseases, serious conditions, and high utilization rates of health services should be registered and classified according to the number and degree of diseases, and frequency of health service utilization. Moreover, tiered compensation and differentiated reimbursement policies can be implemented for different groups. Social security policies should also be improved to subsidize middle-aged and older adults with chronic diseases who live alone and have low-income levels.

## Conclusion

This study selected five major chronic diseases from the top ten global causes of death, published by WHO in 2019, and analysed the middle-aged and older adults aged 45 years and above with the disease. The following conclusions were drawn. First, the implementation of medical insurance policies must be differentiated according to groups of people, especially for those with a high incidence of CHE. Appropriate approaches can be adopted to reduce the premium or increase the reimbursement rate. Second, if the family’s economic burden of disease is high, then their economic conditions can no longer help reduce the likelihood of CHE, which requires accurate identification of health insurance policies. Third, the current medical insurance policy in China does not cover enough imported drugs in the reimbursement of malignant tumour drugs. It is necessary to expand the coverage of drugs, control drug prices, and appropriately improve the reimbursement levels. Fourth, the current medical insurance policy has ignored the reimbursement of outpatient expenses. The burden of drug use for chronic diseases stems from outpatient expenses. Therefore, the medical insurance policy must appropriately increase the reimbursement ratio and increase outpatient expenses’ maximum payment limit.

### Strengths and limitations

This study had some limitations. First, we prioritised the five chronic diseases included in the CHARLS database, which were also among the top ten causes of death published by WHO in 2020. We did not examine other diseases with high incidence in the chronic disease spectrum in China, such as psychiatric disorders. Second, the data used in this study is from the CHARLS database in 2018, which is a cross-sectional study and cannot be compared vertically. Third, due to the incomplete weight value provided by the original database, we could not match the sample after the screening; thus, the sampling weight was not included in the data analysis. In addition, the problem of recall bias in variables such as self-health status has not been solved, which is also a common drawback of retrospective studies.

Nevertheless, the significance of this study mainly lies in the following two points. First, this study targeted a particular group with multiple vulnerable characteristics, namely, middle-aged and older adult families with chronic diseases. Additionally, we conducted multidimensional measurements of the current economic burden of their illnesses, which integrated aspects regarding the economy, their demand and utilization of health services, and different medical insurance systems. To our knowledge, this is almost absent in existing studies. In addition, after achieving the universal coverage of China’s medical insurance system, China faces novel challenges, such as changes in its population structure, the elimination of relative poverty, and the modernization of governance. This study provides evidence on how to improve the financial protection function of the medical insurance system at this stage. It is helpful to understand the current weaknesses of China’s medical insurance system and to capture the characteristics of vulnerable people that should be prioritised in future.

## Data Availability

The datasets analysed during the current study are available in the CHARLS repository, http://charls.pku.edu.cn.

## Abbreviations

CHE: Catastrophic Health Expenditure
WHO: World Health Organization
ECD-family: Elderly Chronic Disease Family
UEBMI: Urban Employee Basic Medical Insurance
URBMI: Urban Resident Basic Medical Insurance
NRCMS: New Rural Cooperative Medical Schemes
URRBMI: Urban and Rural Residents’ Basic Medical Insurance
OOP: Out-of-pocket health expenditure
EXP: Household consumption expenditure
CTP: Household capacity to pay
